# Analysis of novel missense ATR mutations reveals new splicing defects underlying Seckel syndrome

**DOI:** 10.1002/humu.23648

**Published:** 2018-09-24

**Authors:** Marta Llorens-Agost, Janna Luessing, Amandine van Beneden, John Eykelenboom, Dawn O’Reilly, Louise S Bicknell, John J Reynolds, Marianne van Koegelenberg, Matthew E Hurles, Angela F Brady, Andrew P Jackson, Grant S Stewart, Noel F Lowndes

**Affiliations:** 1 Centre for Chromosome Biology, National University of Ireland in Galway, Galway, Ireland; 2 School of Life Sciences, University of Dundee, Dundee, Scotland; 3 Oxford Stem Cell Institute, University of Oxford, Oxford, UK; 4 Department of Pathology, Dunedin School of Medicine, University of Otago, Dunedin, New Zealand; 5 Institute of Cancer and Genomic Sciences, University of Birmingham, Birmingham, UK; 6 Wellcome Trust Sanger Institute, Wellcome Genome Campus, Cambridge, UK; 7 North West Thames Regional Genetics Service, Northwick Park Hospital, Harrow, UK; 8 Institute of Genetics and Molecular Medicine, University of Edinburgh, Edinburg, Scotland

**Keywords:** ATR, chicken, Seckel Syndrome, splicing regulation

## Abstract

Ataxia Telangiectasia and Rad3 related (ATR) is one of the main regulators of the DNA damage response. It coordinates cell cycle checkpoint activation, replication fork stability, restart and origin firing to maintain genome integrity. Mutations of the ATR gene have been reported in Seckel patients, who suffer from a rare genetic disease characterized by severe microcephaly and growth retardation. Here, we report the case of a Seckel patient with compound heterozygous mutations in *ATR*. One allele has an intronic mutation affecting splicing of neighboring exons, the other an exonic missense mutation, producing the variant p.Lys1665Asn, of unknown pathogenicity. We have modeled this novel missense mutation, as well as a previously described missense mutation p.Met1159Ile, and assessed their effect on ATR function. Interestingly, our data indicate that both missense mutations have no direct effect on protein function, but rather result in defective *ATR* splicing. These results emphasize the importance of splicing mutations in Seckel Syndrome.

To maintain genomic integrity, eukaryotic cells have developed a conserved network of pathways of DNA damage sensing, signaling, and repair, known as the DNA Damage Response (DDR). The Ataxia Telangiectasia mutated and Rad3-related (ATR) kinase functions as a master regulator of the DDR in response to the presence of single stranded DNA (ssDNA).

While *ATR* (NM_001184.3) is an essential gene in mammalian cells, hypomorphic mutations have been implicated in the development of Seckel Syndrome (MIM# 210600), a rare human disorder characterized by severe microcephaly, mental retardation, and developmental defects. In 2003, O’Driscoll and colleagues first described a silent homozygous point mutation in exon 9 of *ATR* (c.2101A > G) in a single family, in which the affected patients exhibited a severe form of microcephalic primordial dwarfism (O’Driscoll, Ruiz-Perez, Woods, Jeggo, & Goodship, 2003). Subsequent analysis of this mutation revealed that it disrupted splicing between exons 9 and 10 of the *ATR* gene resulting in the generation of a premature stop codon. Nonetheless, a small amount of normal mRNA was produced at sufficient levels to maintain cell viability (O’Driscoll et al., 2003). Additional mutations in *ATR* were since linked to Seckel Syndrome (Mokrani-Benhelli et al., 2013; Ogi et al., 2012).

In this study, we identified two novel compound heterozygous *ATR* mutations implicated in Seckel Syndrome (Patient P1^ATR^), one of which, c.151+4A > G (NG_008951.1), is known to affect splicing, while c.4995G > T results in an amino acid change, p.Lys1665Asn, of undetermined pathogenicity. Here, we used chicken DT40 cells as a model system to characterize the impact of the p.Lys1665Asn mutation on ATR protein function. Additionally, we modeled ATR protein function from the previously identified p.Met1159Ile missense variant of Patient 27-4BI (Ogi et al., 2012). Interestingly, neither missense mutation significantly affects the function of the ATR protein. However, both exonic point mutations significantly perturb normal splicing of the *ATR* gene, indicating that ATR-Seckel Syndrome is primarily caused by mutations that affect gene splicing.

Patient P1^ATR^ was born to healthy unrelated parents. Intra-uterine growth retardation was noted during the pregnancy. Following birth, the patient exhibited microcephaly, growth retardation, and facial dysmorphia (Figure 1a, Supporting Information Figure S1). Whole exome sequencing of genomic DNA from patient P1^ATR^ identified two novel *ATR* mutations, which were verified by Sanger sequencing (Figure 1c,d). The maternal *ATR* allele of this compound heterozygous patient harbored a mutation in intron 2 (c.151+4A > G), predicted to result in aberrant gene splicing leading to a frameshift at codon 52 and generation of a premature stop 27 bp downstream (p.Ala52Cysfs*9). The paternal ATR allele contains a point mutation located in exon 28 (c.4995G > T) resulting in a p.Lys1665Asn amino acid substitution of unknown pathogenicity (Figure 1c).

Western blot analysis of a fibroblast cell line derived from patient P1^ATR^ confirmed a reduction in total ATR protein expression (Figure 1b), as previously observed in cell lines derived from other Seckel Syndrome patients (Mokrani-Benhelli et al., 2013; O’Driscoll et al., 2003; Ogi et al., 2012). Assessment of DDR activation in cells from patient P1^ATR^ showed a reduction in UV-induced Chk1 phosphorylation (Figure 1e). Consistent with the increased replication stress that is usually seen in cells with defective ATR signaling, cells from patient P1^ATR^ exhibited elevated levels of RPA2 and H2AX phosphorylation following the exposure to UV (Figure 1e). However, the DNA damage-induced phosphorylation of SMC1 and NBS1 in fibroblasts from this patient were relatively unaffected, suggesting that the residual ATR protein retains some normal level of activity toward specific downstream substrates (Figure 1b,e).

Given that both patients (P1^ATR^ and 27-4BI) harbor distinct missense mutations (p.Met1159Ile and p.Lys1665Asn), we tested the effects of these mutations on ATR signaling. To study the p.Met1159Ile and p.Lys1665Asn Seckel mutations, we initially used our previously published chicken DT40 Atr conditional null cell line (Eykelenboom et al., 2013). For this cell line, the equivalent amino acids of the mutated human residues correspond to positions p.Met1180 and p.Lys1685 in the chicken Atr sequence (Figure 1f). Both of these residues are located within regions that are highly conserved between human ATR and chicken Atr, suggesting that these residues could be important for ATR function.

We generated and separately targeted Atr p.Met1180Ile and Atr p.Lys1685Asn cDNA constructs to the Ovalbumin (Ova) locus of AID-Atr conditional-null DT40 cells (Supporting Information Figure S2A and C). Southern blotting confirmed successful targeting of these constructs in AID-Atr DT40 cells (Supporting Information Figure S2B).

Since all *ATR* Seckel hypomorphic mutations previously described result in very low levels of protein expression (Mokrani-Benhelli et al., 2013; O’Driscoll et al., 2003; Ogi et al., 2012), we first examined whether Atr p.Met1180Ile and Atr p.Lys1685Asn mutations caused a reduction in protein stability. Interestingly, the missense *Atr* Seckel mutations had no effect on protein stability in our DT40 system, with both mutant Atr proteins being expressed comparably to the WT control (Figure 1g).

Next, we investigated whether the Atr p.Met1180Ile and Atr p.Lys1685Asn mutations affected cell proliferation. We analyzed proliferation by counting cells every 24 hr in the presence and absence of auxin in the media. Neither Atr p.Met1180Ile nor Atr p.Lys1685Asn mutated cells displayed proliferation deficiencies compared to the WT control (Figure 1h).

To explore the effect of these mutations in the DT40 system more carefully, we assessed whether the mutant cell lines were able to activate ATR in response to hydroxyurea (HU). HU indirectly causes replication fork stalling by depleting the intra-cellular concentration of nucleotides, and ATR activation can be measured by assessing the phosphorylation of Chk1. Our results show that DT40 cells containing Atr p.Met1180Ile or Atr p.Lys1685Asn as their sole source of full-length Atr protein are still able to phosphorylate Chk1 at serine 345 (Figure 1i). We also confirmed normal Atr-dependent signaling by analyzing HU-induced apoptosis (Figure 1j). While loss of functional Atr results in increased apoptosis upon HU treatment (AID-Atr control treated with both AUX and HU), the presence of the Atr p.Met1180Ile and Atr p.Lys1685Asn proteins prevented cells from entering apoptosis.

Our data, obtained using chicken *Atr* cDNA expressed in DT40 cells to model human hypomorphic *ATR* mutations, suggests that the ATR p.Met1159Ile and ATR p.Lys1665Asn missense mutations found in Seckel patients do not affect the function of the ATR protein. This implies that the underlying pathology of these missense mutations might not be related to protein function. However, we cannot exclude the possibility that the missense point mutations destabilize ATR specifically in human cells.

As the Atr p.Met1180Ile and Atr p.Lys1685Asn mutations displayed no effect on Atr function in DT40 cells, we considered the possibility that the equivalent human mutations (ATR p.Met1159Ile and ATR p.Lys1665Asn) may affect splicing by abrogating a splicing enhancer or generating a splicing silencer within their respective exonic sequences. Thus, the phenotype of both mutations could result from exon skipping, rather than from the encoded amino acid change. Therefore, we directly assessed the splicing of exons 18 and 28 in human lymphoblasts (LCLs) derived from patients. Shorter splice variants of the expected sizes were detected by Reverse transcription polymerase chain reaction (RT-PCR) in both patient samples (Figure 2a). In addition, we were still able to detect normally spliced *ATR*, which either corresponds to the equivalent regions of the transcripts from the second *ATR* allele in each patient or to leaky splice mutations. DNA sequencing confirmed that the smaller PCR products correspond to the exon-skipped variants (Figure 2b). In these transcripts either exons 17 and 19 or 27 and 29 are spliced together, resulting in a frameshift and the subsequent generation of a downstream premature stop codon (Supporting Information Figure S3C and D). These truncated forms of the ATR protein (Supporting Information Figure S3A–E) were not detected by Western blotting (data not shown), suggesting that the resulting proteins are either not expressed or unstable. In fact, using cycloheximide to prevent nonsense mediated decay (NMD) of mRNAs with premature stop codons we could establish that skipping of exon 28 from patient P1^ATR^ results in NMD. On the other hand, we could find no evidence for NMD of the mRNA corresponding to skipping of exon 18 from patient 27-4BI.

To confirm exon skipping seen in patient-derived LCLs, we used an *in vivo* splicing assay based on a mini-gene system previously used to investigate splicing regulation of other DDR genes, such as *ATM* and *BRCA1* (Raponi et al., 2014). In this *in vivo* splicing assay, a plasmid containing the *α*-globin gene under a strong promoter was used (Supporting Information Figure S3F). The genomic region of interest was inserted within exon 3 of the *α*-globin gene and this plasmid was then transiently transfected into cells to examine splicing *in vivo*.

We performed our experiments with the control Neurofibromin (NF1) mini-gene obtained from Diana Baralle’s laboratory (Raponi et al., 2014). To analyze the effect of *ATR* c.3477G > T and *ATR* c.4995G > T mutations on exon 18 and 28, respectively, we cloned these exons and flanking intronic regions into the NdeI sites of the NF1 plasmid (Supporting Information Figure S3F). WT and mutant plasmids were transfected into both HeLa and HEK293T cells and RT-PCR was used to assess splicing (Figure 2c).

Although the splicing defects observed in this mini-gene assay were not as striking as those observed in patient cells, we could detect shorter splice variants. These shorter variants corresponded to skipping of either exon 18 or exon 28 in the presence of the *ATR* c.3477G > T and *ATR* c.4995G > T mutations, respectively (Figure 2d).

The Human Splicing Finder (HSF) and SFmap online tools predicted that both mutations, *ATR* c.3477G > T and *ATR* c.4995G > T, could potentially disrupt a binding site for the 9G8 splicing factor within ATR exons 18 and 28, respectively, while potential sites for splicing silencing factors SF2/ASF, hnRNP A1, and Tra2Beta might be created or lost by the presence of the mutations (Supp. Table S6).

Using a previously described 5^′^ end-labeled RNA oligonucleotide probe specific to the 9G8 splicing factor (Gao, Wang, Wang, & Andreadis, 2007) and unlabeled competitor RNA oligonucleotides corresponding to WT or mutant sequences from *ATR* exons 18 and 28, we assessed 9G8 binding in electrophoretic mobility shift assays (Figure 2e). Binding of 9G8 was easily detectable and readily competed by an excess of unlabeled 9G8 probe (Figure 2e, lane 1–3). While the WT *ATR* exon 18 probe did not compete, clear competition was observed when a 1000-fold molar excess of the mutant *ATR* exon 18 probe (c.3477G > T) was used as a competitor (Figure 2e, lane 7). Thus, the c.3477G > T mutation generates a sequence capable of binding the 9G8 splicing factor more efficiently than the WT sequence (Figure 2e). However, in this electrophoretic mobility shift assay, the WT and the mutant competitor RNA oligonucleotides derived from *ATR* exon 28 were indistinguishable (Figure 2f). Thus, the *ATR* c.4995G > T mutation in exon 28 does not have any specific impact upon binding of the 9G8 splicing factor and most likely affects a different splice factor not assessed by the 9G8 bandshift assay.

Seckel Syndrome is a rare genetic disease caused by mutations in several genes, including *ATR* (Mokrani-Benhelli et al., 2013; O’Driscoll et al., 2003; Ogi et al., 2012). In this work, we have characterized two novel compound heterozygous *ATR* mutations in a Seckel Syndrome patient. In one patient, the maternally derived allele harbored a novel intronic mutation in one allele (c.151+4A > G), which is predicted to affect *ATR* splicing; while the second paternal allele carried a missense mutation (c.4995G > T, encoding p.Lys1665Asn) of unknown pathogenicity. However, when we modeled this mutation, along with another Seckel-associated missense mutation (c.3477G > T, encoding p.Met1159Ile) in DT40 cells (Eykelenboom et al., 2013) we found that both *Atr* missense mutations were fully functional. These findings suggested that the missense mutations found in these Seckel patients do not exert their pathology by compromising ATR protein function.

It has previously been shown that the classic *ATR* Seckel mutation (c.2101A > G), which is located within an exon, does not cause an amino acid substitution, but instead affects splicing. The resulting exon skipping in turn results in a frameshift and consequent premature termination of translation (O’Driscoll et al., 2003). In a similar case, a French patient with a mutation in exon 33 of *ATR* was predicted to disrupt a splicing enhancer (Mokrani-Benhelli et al., 2013). Following this trend, it is important to establish whether novel *ATR* Seckel missense mutations, in fact, disrupt the balance between different splicing regulatory elements. Sequences within exons (and introns) are required for successful splicing reactions and loss of these binding sites for a factor that enhances splicing or gaining a site for a factor that silences splicing would be predicted to result in exon skipping (Wang & Burge, 2008).

Therefore, we used human cells derived from patients to further investigate whether the missense mutations c.3477G > T and c.4995G > T (encoding for p.Met1159Ile and p.Lys1665Asn) also affected splicing. Both missense mutations resulted in exon skipping of exons 18 or 28, respectively, in both patient-derived cell lines and when modeled in an ectopic mini-gene-based splicing assay. Thus, our results suggest that while both c.3477G > T and c.4995G > T Seckel mutations alter the amino acid sequence, pathologically they affect mRNA splicing rather than ATR protein function. While we have not fully elucidated the mechanisms behind this splicing deregulation, our *in silico* predictions and biochemical analysis suggest that these mutations might potentially disrupt splicing enhancers or silencers (Supporting Information Table S6). In particular, when using HSF and SFmap online tools, it was predicted that both c.3477G > T and c.4995G > T mutations disrupt a binding site for 9G8 (Supporting Information 2). 9G8 belongs to the serine/arginine (SR) family, comprised several splice factors known to play significant roles in constitutive as well as alternative splicing (Gao et al., 2007). Although SR proteins typically act as splicing activators by binding to exonic enhancers, studies show that in other contexts they can silence splicing (Shen, Kan, & Green, 2004). For example, the 9G8 factor studied here has been reported to promote exon 10 skipping of the Tau protein (Gao et al., 2007). This is reminiscent of our *in vitro* RNA binding studies, in which the c.3477G > T mutation in exon 18 appears to bind the 9G8 splicing factor more effectively than the WT sequence. This data suggests that 9G8 binding suppresses normal splicing and results in exon 18 skipping. On the other hand, and despite the *in silico* predictions, we could not find any evidence that the c.4995G > T missense mutation in exon 28 altered binding of 9G8, indicating that this mutation might affect splicing via a different mechanism (Figure 2f, Supporting Information 2). In fact, *in silico* predictions also suggested that both *ATR* mutations could potentially create a silencer site recognized by the hnRNPA1, while another inhibitory site for Tra2Beta would also be generated in the presence of the c.4995G > T mutation in exon 28 (Supporting Information Table S6). Further studies regarding these factors are necessary to fully understand how these mutations affect ATR splicing.

Taken together, our data combined with previously published observations suggest that *ATR*-Seckel Syndrome is frequently caused by mutations, either exonic or intronic, that disrupt gene splicing. Disease-related missense mutations are often assumed to directly affect protein function, for example, stability or cellular localization. However, there is growing evidence that multiple human disorders caused by exonic mutations, irrespective of any amino change, are, in fact, the consequence of splicing defects (Baralle & Baralle, 2005; Lopez-Bigas, Audit, Ouzounis, Parra, & Guigo, 2005). Interestingly, even nonsense mutations, which are commonly assumed to only disrupt protein function, by generating truncated isoforms, have been shown to cause splicing defects (Cartegni, Chew, & Krainer, 2002).

As a result of exon skipping, both missense c.3477G > T and c.4995G > T mutations are predicted to cause a frameshift and the introduction of a premature stop codon. Our data is consistent with splicing mutations having a bigger impact than previously thought in human disorders (Li et al., 2016; Padgett, 2012). Therefore, new diagnostic techniques, in which genomic screens coupled with whole transcriptome analysis are required to determine whether splicing defects rather than missense mutations underpin disease pathology. This will allow for the proper evaluation of how many human syndromes are caused by splicing mutations, which are currently likely to be under-estimated.

## Supplementary Material

Supplementary material

## Figures and Tables

**Figure 1:**
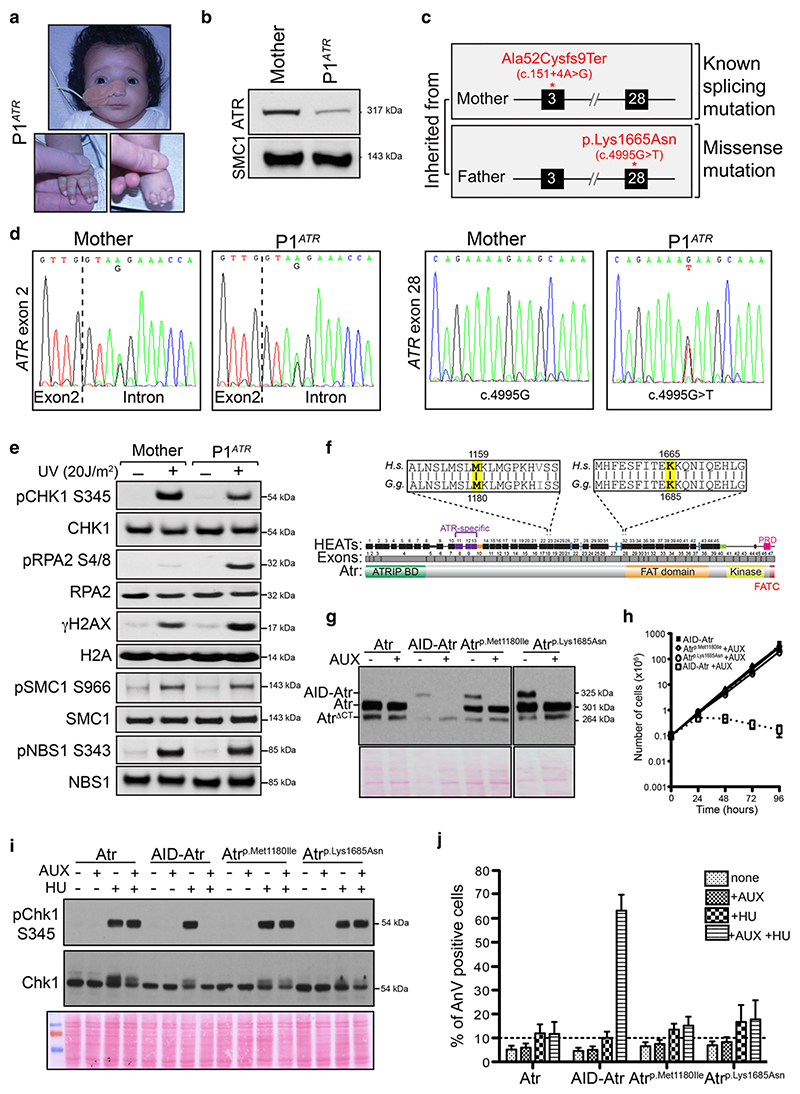
Characterization of the novel p.Ala52Cysfs*9 and p.Lys1665Asn mutations found in patient P1^ATR^ and modeling these mutations in DT40 lymphocytes. (a) Pictures of Seckel patient P1^ATR^. Patient shows typical Seckel features, including microcephaly, dysmorphic facial appearance, and finger/toe abnormalities. (b) Expression of the ATR protein in fibroblasts derived from patient P1^ATR^ and the patient’s mother (paternal cell line not available). ATR levels are analyzed using the ATR-N19 antibody (Santa Cruz). SMC1 is used as loading control. (c) Patient P1^ATR^ harbors compound heterozygous *ATR* mutations, including a known splicing mutation in one allele, plus a novel missense mutation of unknown effects in the other allele. The maternal allele carries a splicing mutation (c.151+4A > G) in intron 2, resulting in skipping of exon 2 and a truncated protein (p.Ala52Cysfs*9). The paternal allele harbors a point mutation in exon 28 (c.4995G > T) that results in a missense mutation (p.Lys1665Asn). (d) Sequencing of ATR exons 2 and 28 from the mother of patient P1^ATR^ and the affected patient. The exon–intron boundaries are indicated with a dashed line, while the mutated bases are shown below the *ATR* sequence. (e) Altered DNA damage signaling in fibroblast cell line derived from patient P1^ATR^. Phosphorylation of the ATR targets pCHK1-S345, pRPA2-S4/8, and *γ*H2AX, as well as pSMC1-S966, pNBS1-S343. Corresponding total protein levels are used as loading control. (f) A linear schematic of chicken Atr is shown, highlighting the HEAT repeat units (black/purple boxes), exons (in gray), and known domains that make up its structure. Overall identity between ATR/Atr proteins (amino acid level) is 77.2%. Residues mutated in patients are shown in yellow, while a single species-specific mismatch eight amino acids C-terminal to Met1180 is shown in gray. (g) Western blot analyses of total cell extracts prepared from DT40 cells expressing Atr, AID-Atr, Atr ^p.Met1180Ile^, and Atr ^p.Lys1685Asn^. Note that AID-Atr untreated sample is under loaded. (h) Growth curve analysis of the Atr ^p.Met1180Ile^ and Atr ^p.Lys1685Asn^ cell lines following treatment with auxin (AUX). Error bars represent the SD from at least three independent experiments. (i) Western analysis of Chk1-S345 phosphorylation. An antibody that recognizes phosphorylation of CHK1 on S345 (Cell Signaling) was used to assess Atr function, while an antibody against total CHK1 (FL-476 Santa Cruz) was used as a loading control. (j) Measurement of HU-induced apoptosis in the indicated cell lines. Error bars in the graph represent the SD from three independent experiments

**Figure 2:**
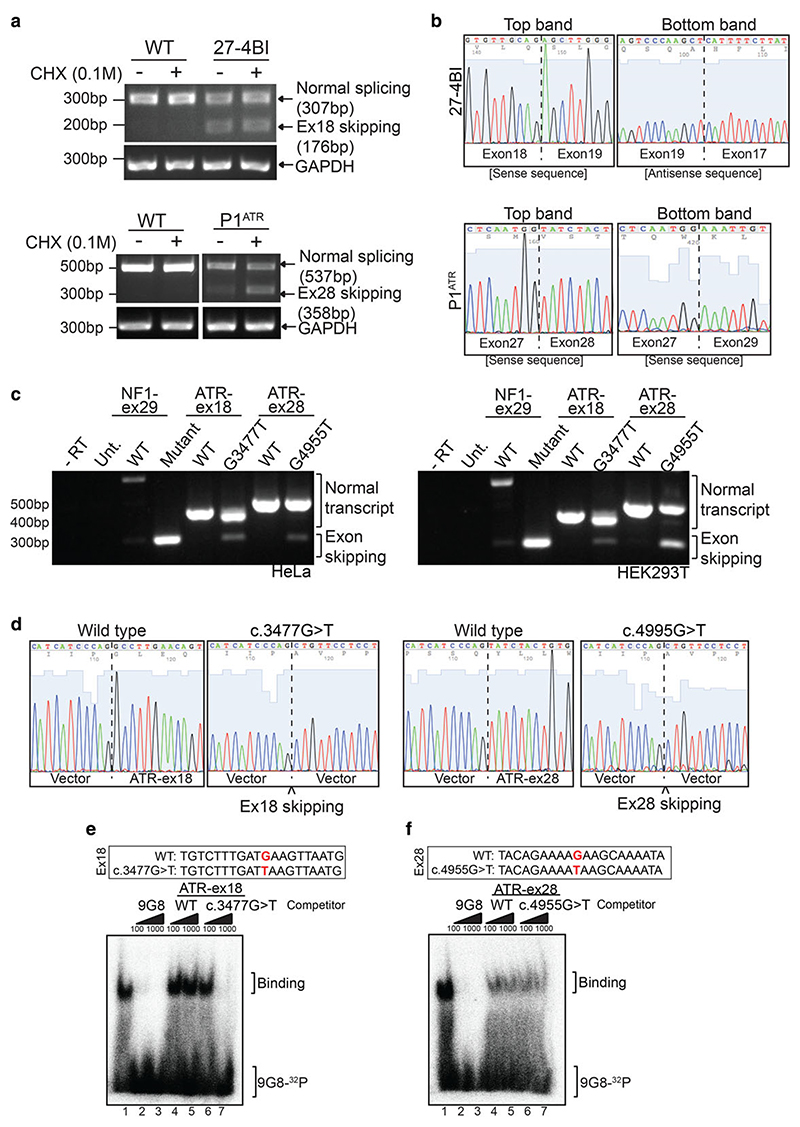
*ATR* exon 18 and 28 skipping in patient 27-4BI and patient P1^ATR^, respectively. (a) PCR analysis of cDNA isolated from LCLs derived from either an unaffected individual (WT) and patients 27-4BI (*ATR* c.3477G > T) or patient P1ATR (*ATR* c.4995G > T). (b) Sequencing results for patient 27-4BI (*ATR* c.3477G > T) and P1ATR (*ATR* c.4995G > T). Upper and lower bands from (a) (no CHX) were sequenced. The chromatogram illustrates exon 18 and exon 28 skipping in the shorter mRNA variants. (c) PCR analysis of the mini-gene splicing assay performed in both HeLa and HEK293T cells. (d) Sequencing results for patient 27-4BI (left panels) and patient P1^ATR^ (right panels). The chromatogram demonstrates absence of exon 18 and exon 28, respectively, upon mini-gene transfection and *in vivo* splicing of *ATR* constructs. (e) Band shift assay using an RNA probe specific to the 9G8 splicing factor and competing RNA oligonucleotides specific to WT and mutated ATR exons 18. (f) Band shift assay using an RNA probe specific to the 9G8 splicing factor and competing RNA oligonucleotides specific to WT and mutated ATR exons 28

## References

[R1] Baralle D, Baralle M (2005). Splicing in action: Assessing disease causing sequence changes. Journal of Medical Genetics.

[R2] Cartegni L, Chew SL, Krainer AR (2002). Listening to silence and understanding nonsense: Exonic mutations that affect splicing. Nature Reviews Genetics.

[R3] Eykelenboom JK, Harte EC, Canavan L, Pastor-Peidro A, Calvo-Asensio I, Llorens-Agost M, Lowndes NF (2013). ATR activates the S-M checkpoint during unperturbed growth to ensure sufficient replication prior to mitotic onset. Cell Reports.

[R4] Gao L, Wang J, Wang Y, Andreadis A (2007). SR protein 9G8 modulates splicing of tau exon 10 via its proximal downstream intron, a clustering region for frontotemporal dementia mutations. Molecular and Cellular Neuroscience.

[R5] Li YI, van de Geijn B, Raj A, Knowles DA, Petti AA, Golan D, Pritchard JK (2016). RNA splicing is a primary link between genetic variation and disease. Science.

[R6] Lopez-Bigas N, Audit B, Ouzounis C, Parra G, Guigo R (2005). Are splicing mutations the most frequent cause of hereditary disease?. FEBS Letters.

[R7] Mokrani-Benhelli H, Gaillard L, Biasutto P, Le Guen T, Touzot F, Vasquez N, Revy P (2013). Primary microcephaly, impaired DNA replication, and genomic instability caused by compound heterozygous ATR mutations. Human Mutation.

[R8] O’Driscoll M, Ruiz-Perez VL, Woods CG, Jeggo PA, Goodship JA (2003). A splicing mutation affecting expression of ataxia-telangiectasia and Rad3-related protein (ATR) results in Seckel syndrome. Nature Genetics.

[R9] Ogi T, Walker S, Stiff T, Hobson E, Limsirichaikul S, Carpenter G, Jeggo PA (2012). Identification of the first ATRIP-deficient patient and novel mutations in ATR define a clinical spectrum for ATR-ATRIP Seckel Syndrome. PLoS Genetics.

[R10] Padgett RA (2012). New connections between splicing and human disease. Trends in Genetics.

[R11] Raponi M, Smith LD, Silipo M, Stuani C, Buratti E, Baralle D (2014). BRCA1 exon 11 a model of long exon splicing regulation. RNA Biology.

[R12] Shen H, Kan JL, Green MR (2004). Arginine-serine-rich domains bound at splicing enhancers contact the branchpoint to promote prespliceosome assembly. Molecular Cell.

[R13] Wang Z, Burge CB (2008). Splicing regulation: From a parts list of regulatory elements to an integrated splicing code. RNA.

[R14] Zheng S, Kim H, Verhaak RGW (2014). Silent mutations make some noise. Cell.

