# Supplementary material for: Analysis of novel missense ATR mutations reveals new splicing defects underlying Seckel syndrome
Source: Hum Mutat. Author manuscript; Available in PMC 2024 Mar 20. (PMC7615757; doi:10.1002/humu.23648)
Supplement: Supplementary material [file EMS194563-supplement-Supplementary_material.docx]

SUPPLEMENTARY MATERIAL

**Supplemental Material 1: Methods**

**Research subjects**

Genomic DNA from affected children and family members was extracted from peripheral blood using standard methods or saliva samples with Oragene collection kits according to the manufacturer’s instructions. Informed consent was obtained from the participating family. Ethics for the studies were approved by the Scottish Multicenter Research Ethics Committee (05/MRE00/74). Maternal consent was provided for the publication of photographs of the affected individual.

**Whole exome sequencing**

Exome sequencing of genomic DNA and variant-filtering was performed as described previously (Martin et al., 2014). Patient genomic DNA re-sequencing was performed by Sanger sequencing of PCR products encompassing exons 2 and 28 of *ATR* with variant-calling using MutationSurveyor (SoftGenetics Inc.) (Suppl. Table 2). Novel mutations have been submitted to the ClinVar database. Preliminary accession numbers are SCV000803364 and SCV000803365 for c.4995G>T and c151+4A>G, respectively.

**Cell Culture**

Chicken DT40 cells were grown at 39.5ºC and 5% CO2 under a humidified atmosphere in RPMI 1640 with L-Glutamine (Gibco or Lonza) supplemented with 10% Foetal Bovine Serum (FBS; Sigma-Aldrich or Lonza), 1% Chicken Serum (CS; Sigma-Aldrich) and 1% Pen/Strep (Sigma-Aldrich or Lonza). Genomic details of the DT40 cell lines used in this study can be found in Supplementary Table 1.

Cell proliferation analysis and stable transfections were carried out as described previously (Eykelenboom et al., 2013). Auxin (IAA; Sigma-Aldrich) was prepared fresh for each experiment at 0.5M in ethanol and used at a final concentration of 0.5mM. Hydroxyurea (HU, Sigma-Aldrich) was prepared at 0.66M in water and used at a concentration of 1mM.

Lymphoblastoid cell lines (LCLs) were generated by immortalising patient-derived peripheral blood mononuclear cells (PBMCs) with EBV. LCLs were cultured at 37ºC in a humidified atmosphere of 5% CO2. The medium used was RPMI 1640 with L-Glutamine (Gibco or Lonza) supplemented with 10% Foetal Bovine Serum (FBS; Sigma-Aldrich or Lonza), 1% L-Glutamine (Sigma-Aldrich) and 1% Pen/Strep (Sigma-Aldrich or Lonza). Patient-derived fibroblasts were derived from a skin biopsy and routinely cultured at 37ºC in a humidified atmosphere of 5% CO2 in DMEM supplemented with 20% FBS (Gibco) and 1% Pen/Strep (Gibco). HeLa and HEK293T cells were cultured at 37ºC in a humidified atmosphere of 5% CO2 in DMEM (Lonza), supplemented with 10% FBS (Sigma or Lonza) and 1% Pen/Strep (Sigma-Aldrich or Lonza).

**Plasmid and DT40 Cell Line Construction**

Details of Ovalbumin-targeting plasmid as well as cDNA expression plasmids of *Atr* and AID-*Atr* can be found in (Eykelenboom et al., 2013). In order to generate the Seckel cDNA expression constructs, the *Atr* cDNA expression vector was used as a template (pLC4) and PCR and side-directed mutagenesis was carried out to introduce the desired point mutations (Suppl. Table 2). Amplification of two PCR products followed by fusion PCR was used to generate the mutated *Atr* constructs. The constructs were then cloned into the pLC4 vector containing the *Atr* cDNA by double SfiI/StuI digestion. The final constructs were then introducted into an Ovalbumin-targeting vector (pJE28). Plasmids containing the *Atr* cDNA mutations were digested with SpeI to release the *Atr* cDNA flanked by promoter and polyA signal; while the Ovalbumin targeting vector was digested with NheI (Suppl. Figure 2).

**Southern Blotting**

Following 0.8% agarose gel electrophoresis of genomic DNA digested with BamHI, standard capillary transfer methods were used and DNA was fixed to a positively charged nylon membrane (Amersham Hybond-N) by UV crosslinking (3000Jm-2 for 1min). Probes for the Ovalbumin gene were PCR amplified from DT40 genomic DNA using the primer pairs Ova_PF3 and Ova_PR3 (Suppl. Table 2) with a Digoxigenin PCR labelling kit (Roche). Hybridisation and detection of the bound probe was carried out using anti-DIG antibody and CSPD (both Roche) according to manufacturer’s instructions.

**Western Blotting**

DT40 cells were lysed at 4°C for 10-30min in Lysis Buffer (50mM Tris, pH 7.5, 150mM NaCl, 0.5% NP40, 1mM EDTA and 5-10% glycerol, supplemented with protease (Sigma) and phosphatase inhibitor cocktails (Roche)). Fibroblasts and LCLs were lysed by sonication in UTB buffer (8M Urea, 50mM Tris pH7.5, 150mM β-mercaptoethanol). Lysates were centrifuged at 13,000-14,000rpm for 10-20min and the supernatant boiled in Laemmli buffer. Samples were then subjected to SDS-PAGE and transferred to a nitrocellulose membrane for protein detection. The following antibodies were used for analysis: ATR-N19 (Santa Cruz), pChk1-S345 (Cell Signalling), CHK1-FL476 (Santa Cruz), pSMC1-S966 (Bethyl Laboratories), SMC1 (Bethyl Laboratories), pNBS1-S343 (Abcam), NBS1 (Genetex), pRPA2-S4/8 (Bethyl Laboratories), RPA2 (Merck Millipore), γH2AX (Merck Millipore) and H2A (Merck Millipore).

**Annexin V/PI Assay**

Cells were seeded at a density of 0.1x106 cells/ml 24h before treatment and subjected to the relevant treatments the following day. About 2x106 cells were resuspended in 50μl of calcium chloride buffer (10mM HEPES pH 7.5, 140mM NaCl, 2.5mM CaCl2) containing 1μl of Annexin V-fluorescein isothiocyanate (FITC) peptide and left at room temperature for 15 min. 300μl of calcium chloride buffer containing 660ng/ml propidium iodide was then added. Samples were measured using a Canto II flow cytometer and analyzed using the BD FACSDiva software.

**RNA extraction and complementary DNA (cDNA) generation**

RNA extraction was performed using TRI reagent (Sigma), while retro-transcription reaction was done following manufacturers’ instructions for High-Capacity cDNA Reverse Transcription Kit (Applied Biosystems).

**Analysis of exon skipping using patient cells**

PCRs to confirm exon skipping were performed using the standard KOD Hot Start DNA Polymerase protocol (Novagen), following RNA extraction from LCL cell lines and generation of cDNA (Suppl. Table 3). PCR products were analysed by DNA electrophoresis in 1.5% agarose TBE gels, gel extracted and sent for sequencing to LGC to confirm exon skipping.

**Generation of mini-gene constructs**

ATR exons 18 and 28 were amplified in separate PCR reactions from human genomic DNA, along with 230 to 300bp of the intronic flanking sequences. Primers used in these reactions carry an NdeI site in their 5’-ends. This restriction site was used to clone the ATR constructs into the NdeI site of the pTB NF1-29 vector (a modified version of the α-globin-fibronectin EDB minigene) obtained from Diana Baralle’s laboratory (Raponi, Buratti, Dassie, Upadhyaya, & Baralle, 2009). Site-directed mutagenesis was then performed to introduce the ATR^p.Met1159Ile^ and ATR^p.Lys1665Asn^ point mutations (Suppl. Table 2).

**Mini-gene assay**

80-90% confluent HEK293T or HeLa cells were transfected with 1μg control (pTB NF1-29) or ATR mini-gene plasmids using Lipofectamine transfection reagent (Invitrogen) according to manufacturer’s instructions. Cells were collected 24h later for RNA extraction and RT-PCR analyses. PCRs to study the mini-gene splicing were carried out using KOD polymerase (Novagen) and primers complementary to sequences in the mini-gene vector flanking the ATR/NF1 constructs (Suppl. Table 2).

**Electrophoretic Mobility Shift Assay (EMSA)**

EMSA reactions were performed in a final volume of 20µl in binding buffer (40mM HEPES, pH 7.9, 50mM KCl, 1mM DTT, 10% glycerol, 0.01% NP40, 0.1mg/ml PolydIdC (P4929, Sigma), 0.1mg/ml BSA, 0.1mg/ml S. cerevisiae tRNA (R8508, Sigma), supplemented with Human Placenta RNAse inhibitor (NEB)). 15µg of HeLa nuclear extract was mixed with 9G8 control or ATR competitor unlabelled RNA oligonucleotides and incubated at room temperature for 5min. 9G8-32P labelled RNA oligonucleotides at 40nM final concentration were then added to the reactions and incubated for another 15min at room temperature. Unlabelled oligonucleotides were used as competitors at 100 and 1000 molar excess compared to the labelled probes (Oligonucleotide sequences can be found in the Supplementary Table 5). Samples were then loaded on a 6% native acrylamide gel in 0.5x TBE buffer and run at a constant 60V for 1.5h on ice. The gel was then frozen and exposed overnight using a Fuji phosphor screen.

**Supplemental Figure 1: Patient description**


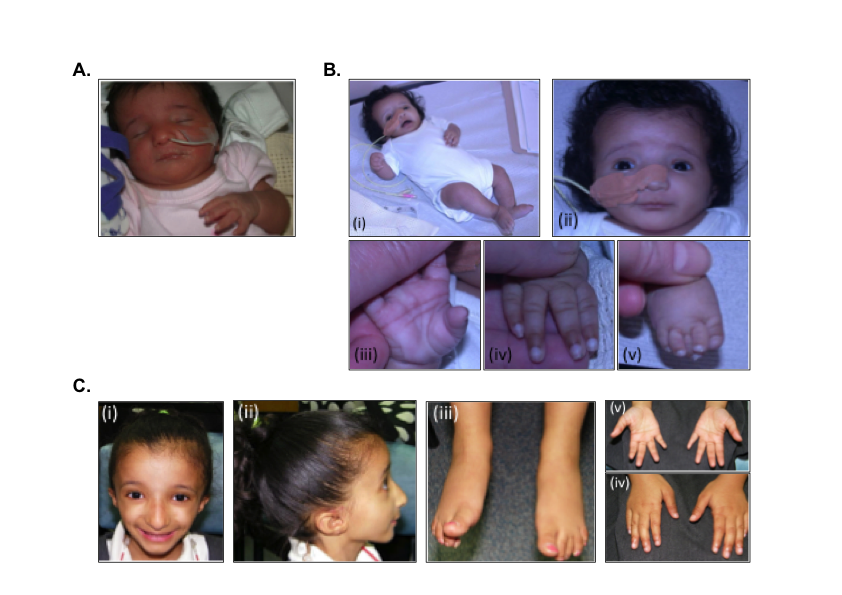


Patient description: At the age of 7 years 5 months her head circumference was 44.6cm, weight 9.35kg and height 88.4cm, all measurements markedly below the 0.4th centile. She had a normal female karyotype, array CGH, echocardiogram and brain MRI scan. Her skeletal survey showed delayed skeletal maturation, some osteopaenia, bilateral coxa vara, partial sacral agenesis and the findings were felt to be consistent with the diagnosis of Microcephalic Primordial Dwarfism (MPD) (Figure 1A, Suppl. Figure 1). She was investigated early on for possible causes of her MOPD phenotype and ATR was considered.

(A) Patient at the age of 3 days. Clinical features include thick dark scalp hair, straight eyebrows, high broad nasal bridge, beaked nose, rhizomelic shortening, tapering fingers and little finger clinodactyly. (B) Patient at 3 months. Clinical features include: rhizomelic shortening (i), thick dark hair, straight eyebrows, hypertelorism, beaked nose and micrognathia (ii) transverse palmar crease (iii) tapering fingers and little finger clinodactyly (iv) short broad hallux and second and fourth toes overlapping the third toe (v). (C) Patient at 7 years and 5 months. Clinical features include: Triangular facies, hypertelorism and small teeth (i), prominent nose and low set posteriorly rotated ears (ii), short fifth toes, broad halluces and second and fourth toes overlap third toes (iii), tapering fingers and little finger clinodactyly (iv), bilateral transverse palmar creases (v).

**Supplemental Figure 2: Generation of *Atr*-mutant DT40 cell lines.**


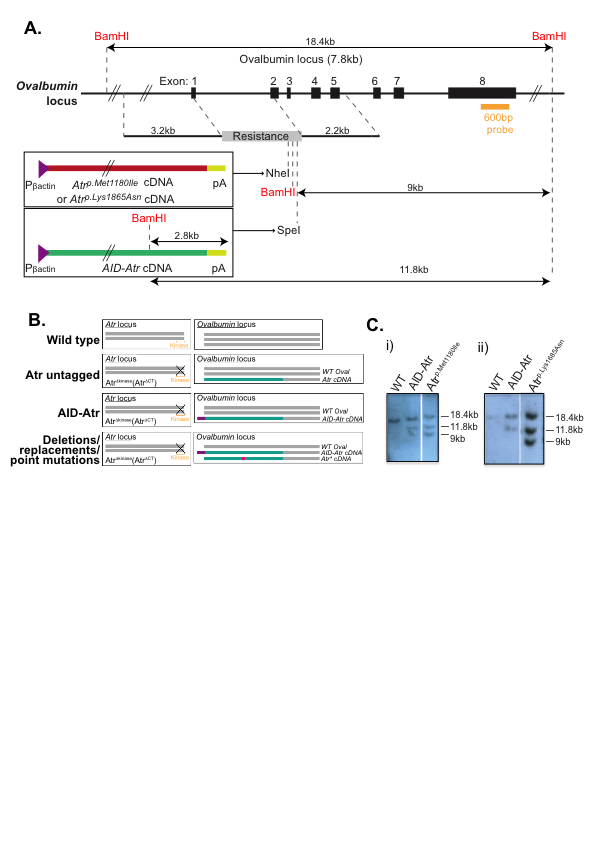


A. Schematic of the *Gallus gallus Ovalbumin* locus, the targeting vector and *AID-Atr* and *Atr* *Seckel* expression cassettes. Relevant restriction sites (as grey dotted lines) and Southern probe binding sites (in orange) are indicated. Relevant BamHI sites (in red) and subsequent fragments expected following targeting of the construct to the *Ovalbumin* locus are also shown. For a non-targeted *Ovalbumin* genomic locus, the expected band is 18.4kb. For *Atr Seckel* cDNAs targeting results in the expected 9kb band; while for the *AID-Atr* cDNA, already present in parental cell line, the expected band is 11.8kb (9 + 2.8 kb). The *Atr Seckel* cDNAs were cloned into the NheI site of the targeting vector, whereas the *AID-Atr* cDNA was introduced in the SpeI restriction site, as reported previously (Eykelenboom et al., 2013). That difference in the cloning site corresponds to a different pattern after the BamHI digest. B. Diagram illustrating the generation of the DT40 cell lines used in this study. The endogenous copies of *Atr* and *Ovalbumin* loci are shown in grey, while the *Atr* cDNAs targeted to the *Ova* locus are shown in light blue. Other features are highlighted as follows: AID tag (purple), Atr mutations (pink) and the Atr kniase domain (yellow) C. Southern screening for correct targeting of cDNA constructs containing *Atr^p.Met1180Ile^* and *Atr^p.Lys1685Asn^* to the trisomic *Ova* locus. The first lane represents wild type cells; the second lane corresponds to the *AID-Atr* parental cell line and the last lane represents targeting to the *Ova* locus of either *Atr^p.Met1180Ile^* or *Atr^p.Lys1685Asn^* cDNA constructs, respectively.

**Supplemental Figure 3:**


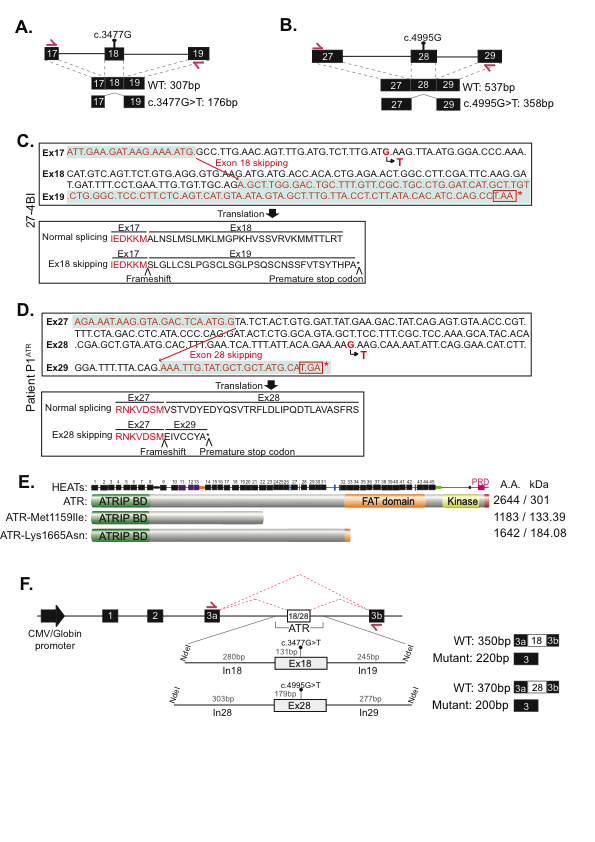


A. Schematic representation of exon18 skipping and PCR analysis of WT and patient 27-4BI (*ATR^c.3477G>T^*) cDNAs. Primers against exon 17 and 19 were used to confirm exon 18 skipping in the presence of the *ATR^c.3477G>T^* mutation. Results shown in the figure correspond to a PCR setup with 27 amplification cycles. B. Schematic representation of exon28 skipping and PCR analysis of WT and patient P1^ATR^ (*ATR^c.4995G>T^*) cDNAs. Primers against exon 27 and 29 were used to confirm exon 28 skipping in the presence of the *ATR^c.4995G>T^* mutation. Results shown correspond to a PCR setup with 29 amplification cycles. C. Effect of the *ATR^c.3477G>T^* mutation at DNA and protein levels. Sequences of exons 17 to 19 are shown in the figure along with part of the corresponding translation for those exons. Presence of the *ATR^c.3477G>T^* mutation causes exon 18 skipping leading to a change in the reading frame and the introduction of a premature stop codon. D. Effect of the *ATR^c.4995G>T^* mutation at DNA and protein level. Sequences of exons 27 to 29 are shown in the figure along with part of the corresponding translation for those exons. Presence of the *ATR^c.4995G>T^* mutation causes exon28 skipping leading to a change in the reading frame and the introduction of a premature stop codon. E. Schematic representation of predicted ATR truncations in the presence of the *ATR^c.3477G>T^* and *ATR^c.4995G>T^* mutations. Predicted sizes of the mutant proteins are shown in both amino acids and kDa. F. Schematic representation *ATR* hybrid mini-genes. Diagram shows the *ATR* genomic regions inserted within exon 3 of the globin gene, along with the location of the mutations studied. Mini-gene exons and introns are indicated as boxes and lines, respectively. Pink arrows indicate primers used to check splicing efficiency by PCR, while dotted lines show the two splicing variants that are expected (normal transcript/exon skipping variant). Length of these PCR products is also shown.

**Supplemental Table 1: Genomic details of DT40 cell lines used in this study**

| **Cell line** | **Genotype** |
| --- | --- |
| WT | WT, Clone 18 ; *Ova ^+/+/+^ ; Atr ^+/+^* |
| Atr | *Ova ^+/+/wt Atr cDNA (PuroR)^; Atr ^ΔCT (HygroR) / ΔCT (BsdR)^ ; Pcmv OsTIR1 ^(G418R)^* |
| AID-Atr | *Ova ^+/+/AID-Atr cDNA (HistoR)^ ; Atr ^ΔCT (HygroR) / ΔCT (BsdR)^ ; Pcmv OsTIR1 ^(G418R)^* |
| Atr^p.^*^Met1180Ile^* | *Ova ^+/ Atr Met1180Ile cDNA (PuroR)/AID-Atr cDNA (HistoR)^ ; Atr ^ΔCT (HygroR) / ΔCT (BsdR)^ ; Pcmv OsTIR1 ^(G418R)^* |
| Atr*^p.Lys1685Asn^* | *Ova ^+/ Atr Lys1685Asn cDNA (PuroR)/AID-Atr cDNA (HistoR)^ ; Atr ^ΔCT (HygroR) / ΔCT (BsdR)^ ; Pcmv OsTIR1 ^(G418R)^* |

**Supplemental Table 2: List of all primers used in this study.**

| **Experiment** | **Description** | **Primer** | **Sequence (5’-3’)** |
| --- | --- | --- | --- |
| Sequencing | PCR products for Sanger sequencing | Exon 2 | F: TCTTGTAAGTTGGTAACATATTCAG  R: TTAAAGAACTCATAGCAAGCAGTAG |
|  |  | Exon 28 | F: TATAATGGCAGAGTTGGAGCAAGAC  R: CTCTCCCCACTAATCTATAAGCTTC |
| Construct generation | Fragment 1 for p.Met1180Ile Seckel mutant | Del_SfiI_F1 | F: CTAGAGCCTCTGCTAACCATGTTC |
|  |  | Point_p.Met1159Ile_R2 [higher GC%] | R: GGGACCCATAAGCTTGATTAAAGACATAAG |
|  | Fragment 2 for p.Met1180Ile Seckel mutant | Point_p.Met1159Ile_F1 [higher GC%] | F: CTTatgtctttaatcaagcttatgGGTCCC |
|  |  | Del_StuI_R2 | R: TGTTCAATTCAGAAATCCATTCAG |
|  | Fragment 1 for p.Lys1685Asn Seckel mutant | Del_SfiI_F1 | F: CTAGAGCCTCTGCTAACCATGTTC |
|  |  | Point_p.Lys1665Asn_R2 [higher GC%] | R: GCTCCTGAATATTTTGTTTGTTCTCTGTGATG |
|  | Fragment 1 for p.Lys1685Asn Seckel mutant | Point_p.Lys1665Asn_F1 [higher GC%] | F: CatcacagagaaCaaacaaaatattCAGGAGC |
|  |  | Del_T7pr_R2 | R: CGTAATACGACTCACTATAGGGCG |
| Sequencing | Verification of Atr constructs | Atr_seq_F1 | F: CATTGCACCTGATGGGTATG |
|  |  | Atr_seq_F2 | F: AGTTTTTGTGAGCGTGCTTG |
|  |  | Atr_seq_F3 | F: ATGCTCCTTGGATTCTGTGG |
|  |  | Atr_seq_F4 | F: CCAAGTGTGCAGTCCTTCAG |
|  |  | Atr_seq_F5 | F: TATTGGAAGGGCATCGAAAG |
|  |  | Atr_seq_F6 | F: CGTATAGGAGAACACTATCAGCAAG |
|  |  | Atr_seq_F7 | F: TTGCAACTGTCCATGAGAGC |
|  |  | Atr_seq_F8 | F: CAGAATGGTCAGCAACTTGG |
|  |  | Atr_seq_F9 | F: TGTGACACGCTTTTTAGACCTG |
|  |  | Atr_seq_F10 | F: GAACAGATTGTGCCCCTTTC |
| Southern Blot | Probe generation | Ova_PF3 | F: TTTATGGGGGAAAAATGCAG |
|  |  | Ova_PR3 | R: CAGATGAGTTGTCCCAGGTG |
| Exon skipping | Study of splicing defects caused by ATRp.Met1159Ile mutation by PCR/Sequencing – Human cell lines | Ex17_fw  Ex19_rv | CATGCAGTTACTGAGCTCTAGTG  GAGGTAGTGGAAGATAGCTGCAG |
|  | Study of splicing defects caused by ATRp.Lys1665Asn mutation by PCR/Sequencing – Human cell lines | Ex27_fw  Ex29_rv | GCAGTTCTAAAGCATGACGATC  CTGGTCTGGTTCTAGCTGAATAG |
| Mini-gene assay | Generation of *ATR-exon18* construct – Mini-gene assay | mini-gene_in17.fw | GACACATATGCTGTTCTAGATGAATACTGGGC |
|  |  | mini-gene _in18.rv | CAGTCATATGGGGAAATAGGTATGTAGGTTC |
|  | Generation of *ATR-exon28* construct – Mini-gene assay | mini-gene_in27.fw | GACACATATGCACCAACAACGTTGTAAGGAAG |
|  |  | mini-gene_in28.rv | CAGTCATATGCACCATCTCCAAGAAGCCATTC |
|  | Introduction of p.Met1159Ile mutation in *ATR-exon18* construct – Mini-gene assay | p.Met1159Ile.fw.sitedm | CAGTTTGATGTCTTTGATTAAGTTAATGGGACCCAAAC |
|  |  | p.Met1159Ile.rv.sitedm | GTTTGGGTCCCATTAACTTAATCAAAGACATCAAACTG |
|  | Introduction of p.Lys1665Asn mutation in *ATR-exon28* construct – Mini-gene assay | p.Lys1665Asn.fw.sitedm | GAATCATTTATTACAGAAAACAAGCAAAATATTCAGG |
|  |  | p.Lys1665Asn.rv.sitedm | CCTGAATATTTTGCTTGTTTTCTGTAATAAATGATTC |
|  | Analysis of mini-gene splicing by PCR | minig_NF1.fw  minig_NF1.rv | CAACTTCAAGCTCCTAAGCCACTGC  TAGGATCCGGTCACCAGGAAGTTGGTTAAATCA |
|  | Sequencing of *NF1*/*ATR* mini-genes | NF1_sequencing | CCACACAGCAAAGAGAAACATAG |

**Supplemental Table 3: Synthetic RNA oligonucleotides used for EMSA**

| Experiment | RNA oligonucleotide | Sequence (5’-3’) |
| --- | --- | --- |
| EMSA | 9G8 | GUACCUUCACACGUCCCAUG |
|  | ATR_exon18_WT | UGUCUUUGAUGAAGUUAAUG |
|  | ATR_exon18_Met1559Ile | UGUCUUUGAUUAAGUUAAUG |
|  | ATR_exon28_WT | UACAGAAAAGAAGCAAAAUA |
|  | ATR_exon28_Lys1665Asn | UACAGAAAACAAGCAAAAUA |

**Supplemental Material 2: *In silico* prediction of potential splicing sites**

To investigate whether the *ATR* mutations could potentially affect any splicing regulatory motif, two online prediction tools were used: The Human Splicing Finder (HSF) and Splicing Factor map (SFmap). As suggested by Vihinen et al (2013), more than one method was used and the results were combined to have a broader view of the potential sites affected. Besides, the parameters and program options are indicated below.

**Supplemental Table 4. Software description.**

| Programme | Version | Prediction | Reference |
| --- | --- | --- | --- |
| Human Splicing Finder (HSF) | 3.0 | Intronic and exonic splicing motifs, as well as binding sites for hnRNP A1, Tra2-β and 9G8 splice factors. | Desmet, F.-O., Hamroun, D., Lalande, M., Collod-Béroud, G., Claustres, M., & Béroud, C. (2009). Human Splicing Finder: an online bioinformatics tool to predict splicing signals. Nucleic Acids Research, 37(9), e67. http://doi.org/10.1093/nar/gkp215 |
| SF map | 1.8 | Multiple known splice factor binding sites. | Paz, I., Akerman, M., Dror, I., Kosti, I., & Mandel-Gutfreund, Y. (2010). SFmap: a web server for motif analysis and prediction of splicing factor binding sites. Nucleic Acids Research, 38(Web Server issue), W281–W285. http://doi.org/10.1093/nar/gkq444 |

In both cases, WT and mutated exonic sequences were examined separately. The results were then analysed by manually comparing the WT and mutant outputs, focusing particularly in the position of interest. Specifically, for the mutation in exon 18, it was investigated whether any binding site was created or disrupted by the presence of the *ATR* *^c.3477G>T^* mutation at position 27, when comparing the WT and mutant results. On the other hand, for the mutation in exon 28, position 143 was closely looked at, to study if any splice factor binding motif was affected by the presence of the *ATR* *^c.4995G>T^* mutation.

When using the HSF online tool, WT and mutated sequences were evaluated separately (by choosing ‘Analysis of sequence’) and pasted manually into the software. Once the results were retrieved, we evaluated the results under ‘Tables’, tabs ‘ESE motifs from HSF’ and ‘hnRNP motifs’, which predict binding motifs for splice factors that promote and downregulate splicing, respectively. Although more information could be obtained from other sections in this online tool, these were considered the most informative, as potential candidates for binding assays, such as EMSA, were suggested.

When analysing the effects with SFmap, individual sequences were again pasted into the software and results were visualized in the ‘Motif prediction summary’ section.

**Supplemental Table 5. Input sequences.**

Sequences of *ATR* exons 18 and 28 were analysed using the programs stated in the previous table. Length of exons and position of mutations of interest are indicated. Such mutations are shown in bold and underlined within the sequence.

| Exon | Status | Sequence |
| --- | --- | --- |
| *ATR-18* (131bp; mutation in position 27) | WT | GCCTTGAACAGTTTGATGTCTTTGAT**G**AAGTTAATGGGACCCAAACATGTCAGTTCTGTGAGGGTGAAGATGATGACCACACTGAGAACTGGCCTTCGATTCAAGGATGATTTTCCTGAATTGTGTTGCAG |
|  | Mutant: c.3477G>T | GCCTTGAACAGTTTGATGTCTTTGAT**T**AAGTTAATGGGACCCAAACATGTCAGTTCTGTGAGGGTGAAGATGATGACCACACTGAGAACTGGCCTTCGATTCAAGGATGATTTTCCTGAATTGTGTTGCAG |
| *ATR-28* (179bp; mutation in position 143) | WT | TATCTACTGTGGATTATGAAGACTATCAGAGTGTAACCCGTTTTCTAGACCTCATACCCCAGGATACTCTGGCAGTAGCTTCCTTTCGCTCCAAAGCATACACACGAGCTGTAATGCACTTTGAATCATTTATTACAGAAAA**G**AAGCAAAATATTCAGGAACATCTTGGATTTTTACAG |
|  | Mutant:  c.4995G>T | TATCTACTGTGGATTATGAAGACTATCAGAGTGTAACCCGTTTTCTAGACCTCATACCCCAGGATACTCTGGCAGTAGCTTCCTTTCGCTCCAAAGCATACACACGAGCTGTAATGCACTTTGAATCATTTATTACAGAAAA**T**AAGCAAAATATTCAGGAACATCTTGGATTTTTACAG |

**Supplemental Table 6. Predicted splice sites.**

Potential sites for binding of splice factors that would be affected (created or disrupted) by the *ATR* Seckel mutations are shown here.

| Mutation | Prediction tool | Site disrupted | Site created |
| --- | --- | --- | --- |
| *ATR* *^c.3477G>T^* | HSF | 9G8 | hnRNP A1 |
|  | SFmap | SF2/ASF | - |
| *ATR ^c.4995G>T^* | HSF | 9G8 | hnRNP A1 |
|  | SFmap | Tra2Beta | - |
